# HSP90 inhibitor, celastrol, arrests human monocytic leukemia cell U937 at G0/G1 in thiol-containing agents reversible way

**DOI:** 10.1186/1476-4598-9-79

**Published:** 2010-04-16

**Authors:** Bin Peng, Limin Xu, Fanfan Cao, Tingxuan Wei, Chunxin Yang, Georges Uzan, Denghai Zhang

**Affiliations:** 1 Sino-French Cooperative Central Lab, Shanghai Gongli Hospital, 207 Ju Ye Road, Pudong New District, Shanghai, 200135, China; 2 Pharmaceutical Department, Zhong Shan Hospital, Shanghai Fudan University, 136 Yi Xue Yuan Road, Shanghai, 200032, China; 3 U972, Inserm, Bâtiment Lavoisier, Hôpital Paul Brousse, 12 avenue Paul Vaillant Couturier, 94807 Villejuif Cedex, France

## Abstract

**Background:**

Because some of heat shock protein 90's (HSP90) clients are key cell cycle regulators, HSP90 inhibition can affect the cell cycle. Recently, celastrol is identified both as a novel inhibitor of HSP90 and as a potential anti-tumor agent. However, this agent's effects on the cell cycle are rarely investigated. In this study, we observed the effects of celastrol on the human monocytic leukemia cell line U937 cell cycle.

**Results:**

Celastrol affected the proliferation of U937 in a dose-dependent way, arresting the cell cycle at G0/G1 with 400 nM doses and triggering cell death with doses above 1000 nM. Cell cycle arrest was accompanied by inhibition of HSP90 ATPase activity and elevation in HSP70 levels (a biochemical hallmark of HSP90 inhibition), a reduction in Cyclin D1, Cdk4 and Cdk6 levels, and a disruption of the HSP90/Cdc37/Cdk4 complex. The observed effects of celastrol on the U937 cell cycle were thiol-related, firstly because the effects could be countered by pre-loading thiol-containing agents and secondly because celastrol and thiol-containing agents could react with each other to form new compounds.

**Conclusions:**

Our results disclose a novel action of celastrol-- causing cell cycle arrest at G0/G1 phase based upon thiol-related HSP90 inhibition. Our work suggests celastrol's potential in tumor and monocyte-related disease management.

## Background

Cancer therapy targeting HSP90 has shown great promise [1,2]. A wide range of oncogenic client proteins crucial for oncogenesis are stabilized, matured by, and thus dependent on HSP90. The harsh environmental conditions found in tumors, such as hypoxia and low pH, as well as outside factors, such as poor nutrition, tend to destabilize proteins and further their dependence on HSP90. This hypothesis is supported by the higher HSP90 levels found in tumor cells, which can comprise as much as 4-6% of cellular proteins in contrast to the 1-2% seen in normal cells [3,4]. When used as a single agent or in combination with chemotherapy, HSP90 inhibitors have shown anti-tumor effects in cellular studies, animal model studies, and clinical evaluations [5-7]. However, it is too early for many of these inhibitors or their derivatives to have received Food and Drug Administration approval. In this sense, research on novel HSP90 inhibitors is attractive.

Natural substances are often key components of HSP90 inhibitors [8]. After geldanamycin, a natural product isolated from the bacteria Streptomyces hygroscopicus, was found to be an HSP90 inhibitor, a variety of natural HSP90 inhibitors have been identified. Among these are herbimycin, radicicol, novobiocin, coumermycin A1, clorobiocin, epigallocatechin gallate, taxol, pochonin, derrubone, gedunin, and the more recently identified celastrol [2]. Celastrol, also called tripterine, is a quinone methide triterpenoid isolated from the Chinese plant Tripterygium wilfordii Hook F (TWHF), which has been used as an anti-rheumatic in China for many years. Celastrol can activate HSF1, induce expression of some HSPs [9,10], down-regulate HSP90's ability in binding to ATP [11], and disrupt the combination of HSP90 with co-chaperone Cdc37 [12]. All these effects indicate inhibition of HSP90 activities. In agreement with data on the anti-tumor effects of other HSP90 inhibitors, celastrol showed similar action upon a variety of tumor cells [11-15]. Moreover, using *in silico *screens of public gene expression data, celastrol has recently been discovered to eradicate acute myelogenous leukemia stem cells through simultaneous inhibition of NF-κ B-mediated survival signals and induction of oxidative stress [16]. It is therefore possible that when compared to other HSP90 inhibitors celastrol possesses unique anti-tumor properties.

Anti-tumor effects can be achieved by cell death and/or cell cycle arrest. Until now, most reports attributed celastrol's anti-tumor effects on its death-triggering action, leaving celastrol's effects on cell cycle almost unexplored. Some key cell cycle regulator proteins, such as Cdk4 and Cdk6, are clients of HSP90 (see the website http://www.picard.ch for further details), and the partner/activator of these Cdks, Cyclin D1, is also affected by the HSP90 molecular chaperone. It has been proven that HSP90 inhibitors can affect these Cdks and Cyclin D1, causing cell cycle arrest [17,18]. As a novel inhibitor of HSP90, celastrol might also affect these proteins and cause cell cycle arrest, an issue that needs to be adequately addressed to fully understand celastrol's anti-tumor effects.

In this study, we observed the effects of celastrol on proliferation in human monocytic leukemia cell line U937. The results showed that in addition to quickly inducing apoptosis at high doses, celastrol could also arrest cells at G0/G1 phase at lower dosages. These effects were accompanied by elevation of HSP70 levels, down-regulation of Cyclin D1 and Cdk4, and a reduction in HSP90/Cdc37/Cdk4 complex levels. All of these actions could be reversed by pre-treatment of cells with small thiol-containing molecules, whereas non-thiol anti-oxidant agents could not reverse celastrol's effects. Chemical reaction confirmed celastrol's interaction with the thiol group. Our study discloses celastrol's novel action on the cell cycle and sheds additional light on the working mechanisms of this agent.

## Results

### Antiproliferative effects of celastrol

**Figure 1:**
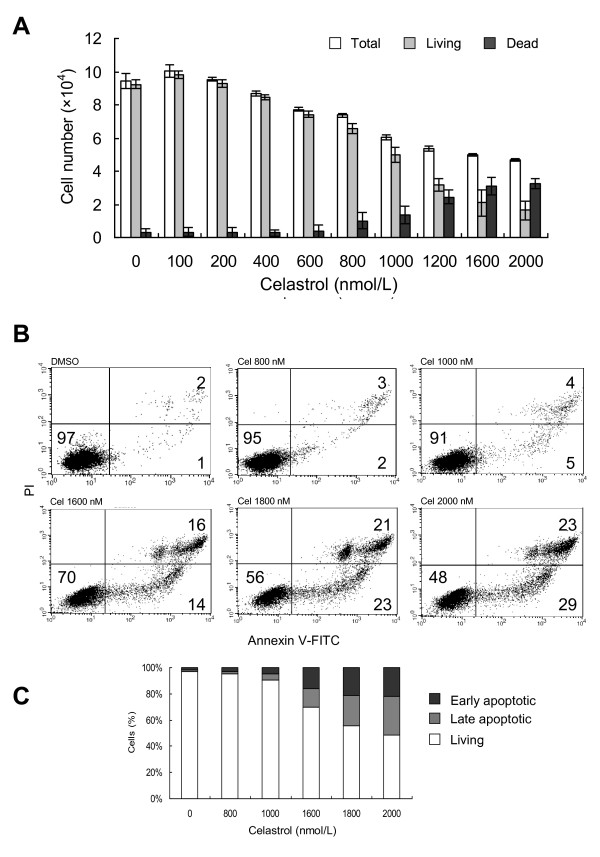
**Effects of celastrol on U937 culture proliferation and viability *in vitro***. Cells were seeded at 2 × 10^5^/ml in 96-well culture plate and treated with indicated doses of celastrol for 1 d. A: Dose-dependent effects of celastrol on the number of total, living and dead cells. After treatment, the numbers of living and dead cells in each sample was determined by single-tube platform flow cytometry using self-made cell-Beads as an internal standard (detailed in *Methods*). Each data point represents the mean value of three test repetitions. B: Dot plot for flow cytometric analysis of living and apoptotic cells. The samples were labeled with Annexin V- FITC and PI double staining, and then detected by FCM. Living cells tested negative for both Annexin V and PI. Populations testing Annexin V positive/PI negative were in early-stage apoptosis, and double positives were in late-stage apoptosis. The percentages of each population are labeled in the corresponding plot region. C. Bar graph showing percentage of living, early, and late-stage apoptotic cells in samples with varying treatments. The values shown are the mean of three independent experiments.

U937 cellular numbers were determined by FCM based on a modified one-tube platform, which can accurately count the number of total, living, and dead cells in a sample [19,20]. After being cultured for 1 day *in vitro*, untreated U937 demonstrated rapid proliferation, with cellular numbers about 2.4 times the initial quantity. As shown in Figure 1A, when the final dosage of celastrol used reached 400 nM, U937 numbers began decreasing compared to the untreated control; as dosages increased further, cell numbers gradually decreased. At 1600 nM, U937 quantities were almost the same as the original numbers.

Further analysis disclosed two ways in which celastrol reduced cell numbers. At dosages ranging from 400 nM to 800 nM, the total number of cells (living and dead) decreased, but dead cell numbers remained constant. Therefore, within this range, cell division disruption was the main reason for cell number reductions. With doses of 800 nM and higher, dead cells increased with dose increases (Figure 1A). This implies an additional cause of proliferation inhibition - i.e.: celastrol began to cause cell death. In fact, a dose-driven rise in apoptotic rates at doses above 1000 nM was revealed by flow cytometric (FCM) analysis of Annexin V/PI stained cells (Figure 1B and 1C).

### Cell cycle arrested at G0/G1 by celastrol

**Figure 2:**
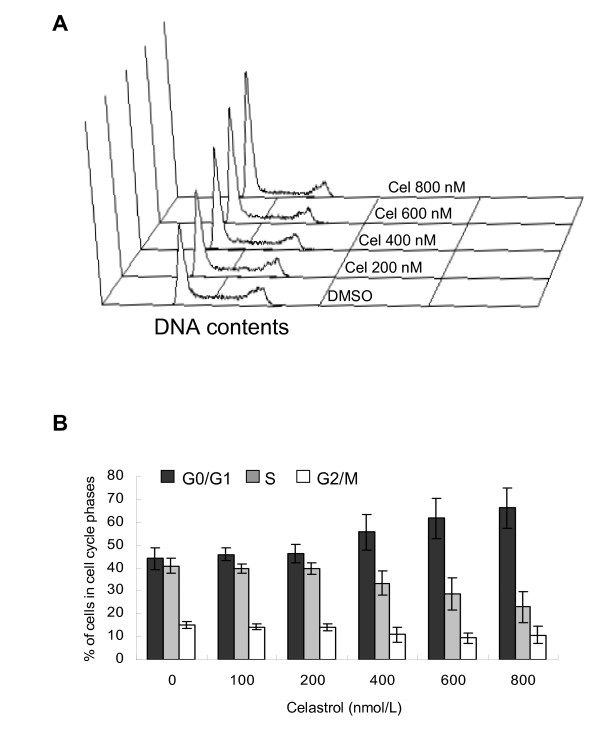
**Celastrol arrests cell cycle at G0/G1 phase in U937**. Following the treatment, cells were incubated with RNase A and PI, cell cycle was assayed by FCM. A: Panel of flow cytometric histograms showing DNA content in samples treated with various doses of celastrol. X-axis represents DNA content and Y-axis shows cell number. B: Percentages of sample cells in each cell cycle phase. The dose-effects of celastrol on the percentages of U937 cells at each phase of cell cycle are shown. Each value is found as the mean of three independent experiments.

Cell cycle was determined by FCM based on PI staining of DNA contents. U937 cells demonstrated a normal diploid distribution in the untreated control, showing fast proliferation characteristics; with cells in S + G2/M accounting for more than half of the cell total. Celastrol at 400 nM reduced cellular numbers as mentioned above and caused a correspondent increase in the number of cells at G0/G1, a change that continued until dosage reached 800 nM (Figure 2). The maximum dose used in this study was 800 nM, as samples treated with doses above 800 nM had large dead cell populations that interfered with accurate calculation of cell cycle location.

### Reducing the levels of Cyclin D1, Cdk2, Cdk4, and Cdk6 in U937

**Figure 3:**
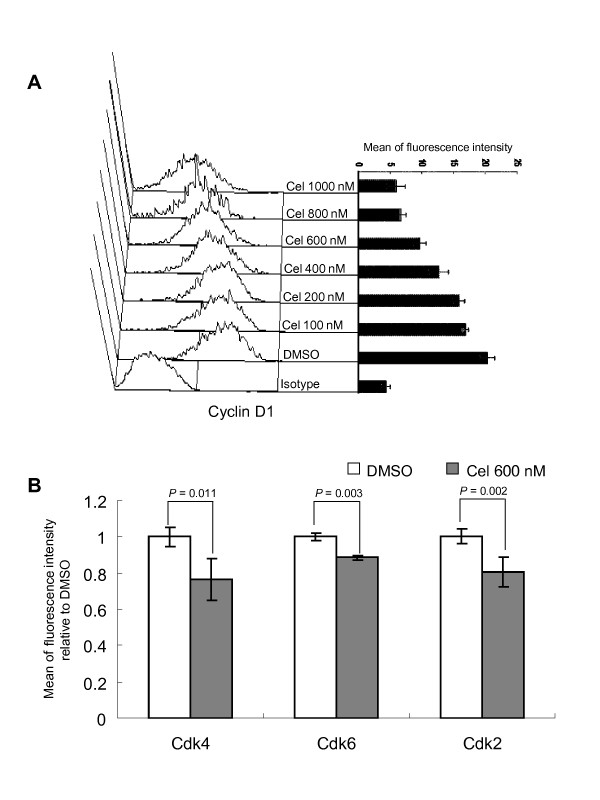
**Celastrol decreases the level of Cyclin D1 and some Cdks**. Following treatment, cells were incubated with indicated antibodies and the expressions of proteins were detected by FCM detailed in *Methods*. A: Celastrol induces reduction of Cyclin D1 in a dose-dependent manner. The left panel shows the histogram for FCM detection of Cyclin D1 expression with X-axis as fluorescence intensity and Y-axis representing cell number. The right panel shows the detected intensities of this protein. Each value represents the mean of three independent experiments. B: Effects of celastrol on Cdk4, Cdk6, and Cdk2 expressions. After exposure to 600 nM celastrol for 1 d, the proteins were detected by FCM. Y-axis represents the relative levels of each protein in different treatments, with the protein level in DMSO-control sample being set at 1.0. Each value is the mean of three independent experiments.

Cyclin D1 and some Cdks are important to cell cycle progress at G1 phase. To examine whether the observed G0/G1 arrest was related to changes in these proteins, we tested some of them by FCM. The results showed that all cells, treated or not, were positive for Cyclin D1, Cdk4, Cdk6, and Cdk2. FCM detection showed that Cyclin D1 was down-regulated by celastrol in a dose-dependent way (Figure 3A). Antibody labelling of Cyclin D1 in conjunction with 7-AAD staining of DNA content was performed to determine the relationship between Cyclin D1 reduction and different cell cycle phases. The results showed that the reduction of this protein was not related to the cells' location in cycle, as the cells at G0/G1, S, and G2/M phases all displayed similar degrees of Cyclin D1 expression decrease (data not shown).

Of the three Cdks detected, Cdk4 was reduced most greatly, while Cdk2 and Cdk6 levels were less affected by celastrol (Figure 3B). It has been reported that Cdk4 is more sensitive to HSP90 inhibitors than Cdk2 and Cdk6 [17]. Since Cdk4 acts during the early G1 stage, while Cdk6 and Cdk2 acting sequentially later, celastrol-caused arrest likely begins at the onset of G1. As was the case with Cyclin D1, the reductions of Cdk4, Cdk6, and Cdk2 levels were unrelated to the cells' location in cycle (data not shown).

### Elevation of HSP70, inhibition of HSP90's ATPase activity, and disruption of the HSP90/Cdc37/Cdk4 Complex in U937 by Celastrol

Since celastrol is reported to inhibit HSP90 activity in several cellular models [11,12,21], and Cdk4 and Cdk6 are clients of HSP90, we hypothesized Cdks reduction in our model might be related to HSP90 activity. To test this hypothesis, we first detected celastrol's effect on HSP90 activity and then determined HSP90/Cdc37/Cdk4's levels.

**Figure 4:**
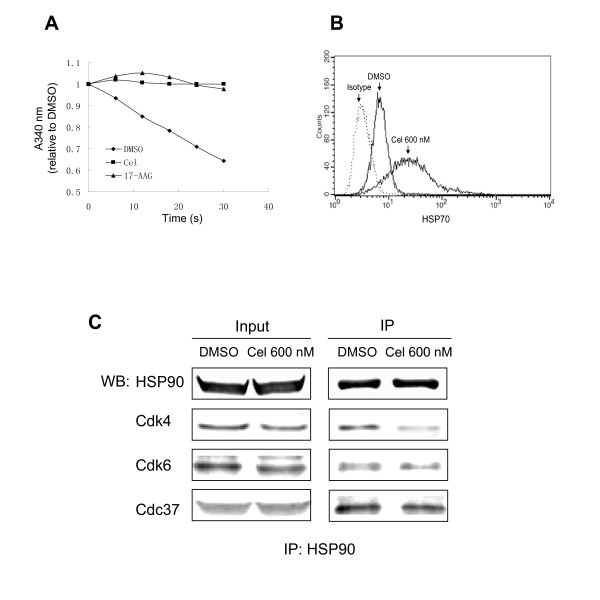
**Effects of celastrol on HSP90's ATPase activity, HSP70 expression and the HSP90-Cdc37-Cdks complex**. A: Celastrol inhibits the activity of ATPase. Co-immunoprecipition of HSP90 was performed on untreated cells, and the beads-bound immunoprecipites were separated into three equal portions before incubation with celastrol, 17-AAG, or DMSO. ATPase activity was determined as the decrease of the absorbance at 340 nm, detailed in *Methods*. B: Celastrol induces the increase of HSP70. Cells treated with 600 nM celastrol for 1 d and HSP70 levels were deteceted by flow cytometry, as detailed in *Methods*. X-axis shows channel number and Y-axis shows cell number. C: Celastrol disrupts the HSP90/Cdc37/Cdks complexes. Cells treated with 600 nM celastrol for 1 d, then were used for immunoprecipitation by anti-HSP90 (H9010), detailed as in *Methods*. The left column (input) displays the detection based on total proteins of cells, while the right column (IP) shows detection of immunoprecipitation using the anti-HSP90 (H9010) antibody.

A regenerating coupled enzyme assay [22] was used to observe celastrol's effects on ATPase activity in the protein complex pulled-down by anti-HSP90. We found that the ATPase activity in this HSP90-containing complex was inhibited by celastrol (Figure 4A) (Inhibition of ATPase activity in the HSP90 complex was also confirmed by a different ATPase assay method, see additional file 1). Additionally, as shown in Figure 4B, expression of HSP70 elevated nearly 4.5 fold when 600 nM of celastrol was used. Since induction of HSP70 is indicative of HSP90 inhibition, we believe that celastrol exerted HSP90-inhibiting activity in our model.

Then we observed celastrol's effects on HSP90/Cdc37/Cdk4 complex. As seen in Figure 4C, the protein complex immunoprecipitated by the anti-HSP90 antibody showed lower levels of Cdk4 and Cdk6, indicating that HSP90/Cdc37/Cdk4 complex formation was disrupted by celastrol. The results showed that the Cdc37 co-chaperone levels in the complex were also decreased, this result is consistent with the previous report that celastrol disrupted Cdc37-HSP90 interaction [12].

### Free thiol-containing agents prevented the effects of celastrol

**Figure 5:**
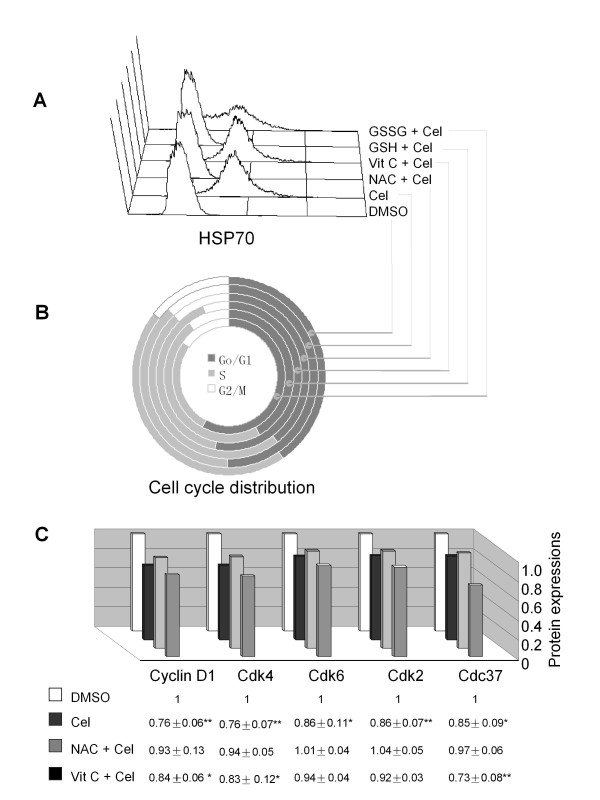
**Reversing effects of thiol-containing agents on the actions of celastrol in U937**. Cells were seeded at 2 × 10^5^/ml in a 24-well plate. After being pre-treated with 2 mM NAC, 0.1 mM Vit C, 2 mM GSH or 2 mM GSSG for 1 h, cells were exposed to 600 nM celastrol for 1 d. At the end of the indicated time points, cells were analysed for cell cycle location and protein detection by FCM. A: Flow cytometric histogram of HSP70 expressions in U937 with various treatments. B: Cell cycle distributions of drug treated U937. The dark gray, light gray and white areas in the circle track represent the ratio of cells at G0/G1, S, and G2/M phases, respectively. C: Expressions of selected proteins in U937 with different pre-treatments. The vertical axis represents the relative levels of each protein, which were determined by dividing individual protein intensity by its levels in the DMSO-treated sample. The DMSO-control sample was set at 1.0. The plotted data represents the mean results of three repetitions. The values under the figure are mean ± SD for each sample. * *P *< 0.05, **P < 0.01 when compared with DMSO-control.

Other models have reported that thiol can reverse the action of celastrol [9], so we tested thiol in our model. The results showed that pre-treatment with the thiol-containing agent N-acetylcysteine (NAC) 1 h before loading celastrol could effectively reverse celastrol's cell cycle arresting action. However, the non-thiol reducing agent we tested, vitamin C (Vit C), did not work in this way. Other thiol-containing agents, such as reduced glutathione (GSH), also showed reversing effects, while oxidized glutathione (GSSG), an agent containing sulfur but without free thiol, did not exhibit countering effects (Figure 5B). Accordingly, elevated levels of HSP70 and down-regulation of Cdks, Cyclin D1, and Cdc37 by celastrol were reversed by NAC but not by Vit C (Figure 5A and 5C).

### Free thiol-containing amino acid synthesis with celastrol via chemical reaction

**Figure 6:**
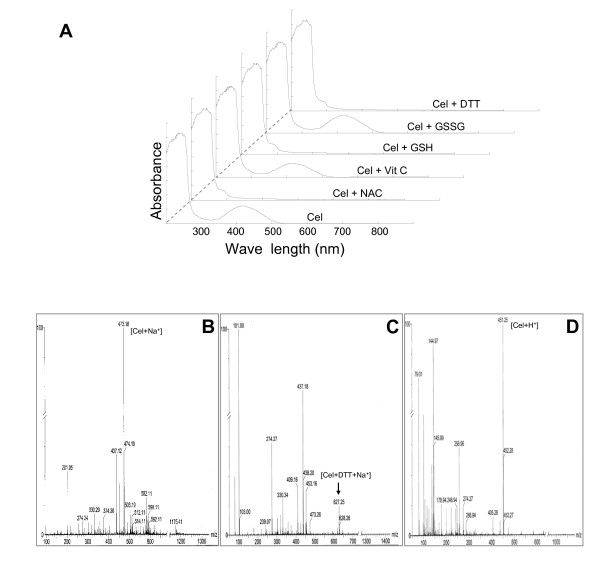
**Reactions between celastrol and thiol-containing agents and between celastrol and non-thiol small molecules**. A: Absorption spectra of celastrol mixed with different agents. Celastrol was mixed with different small molecules in ratio of 1:2, the absorption spectra then detected. B-D: Mass spectrum analysis of the following agents or reactions: (B) celastrol; (C) the mixture of celastrol and DTT at a 1:2 molar ratio in DMSO; (D) celastrol and DTT reaction result with formic acid added (to save space, the detection patterns presented have been truncated. The non-truncated view can be found in this manuscript's additional file 2).

To find out the reason for free thiol-containing agents' reversing effect, we tested if celastrol could directly react with thiol. When celastrol was mixed with the thiol-containing agents, NAC, GSH, or Dithiothreitol (DTT), celastrol's colour disappeared. This colour change was not seen when celastrol was mixed with Vit C or GSSG. When excited by UV light, celastrol showed an absorbance peak at 440 nm which disappeared when incubated with free thiol-containing agents (NAC, GSH or DTT). The absorbance peak was unaffected when incubated with GSSG or non-thiol reducing agent Vit C (Figure 6A). To further confirm a celastrol-thiol reaction, we performed mass spectrum (MS) analysis on the addition compounds formed by celastrol and thiol- (non thiol-) containing agents. The observed m/z 473.18 in the sample of celastrol alone in DMSO was consistent with predication of celastrol (MW 450) plus one natrium ion (Figure 6B). The calculated mass of celastrol plus DTT and one natrium agreed well with the observed m/z 627.25, indicating that these two molecules reacted and produced a new compound with a larger mass (Figure 6C, see additional file 2 for non-truncated view). Similar results were seen when celastrol was mixed with other thiol- containing agents, such as NAC or GSH. Evidence of reaction was not seen in celastrol and non-thiol small molecules (Vit C or GSSG) mixtures (data not shown). Interestingly, after adding an amount of formic acid (FA) to the celastrol and DTT combination, celastrol's orange-red color reappeared and mass spectrum analysis showed m/z 451.25 (Figure 6D). This reading is consistent with calculated mass of celastrol plus one hydrogen ion, and thus indicates that the adduct reactions between celastrol and thiol-containing agents are reversible under acidic conditions. ^1^H NMR analysis further indicated that H6 in celastrol's ring B might be the location for reactions (see additional file 3).

## Discussion

In this study, we investigated the effects and relevant mechanisms of celastrol on human monocytic leukemia cell line U937 proliferation. Celastrol's proven effectiveness in anti-tumor treatments is thought to be primarily due to apoptosis induction [12-15,23,24]. However, we disclosed that in addition to causing cell death, this agent could also arrest the cell cycle at G0/G1. This novel action was accompanied by and related to down-regulation of Cyclin D1 and its partners Cdk4, Cdk6, and Cdk2. The interaction between Cyclin D1 and its partners, especially Cdk4, is vital during the G1 phase, and disruption of either Cyclin D1 or Cdk4 can induce G0/G1 arrest [25-27].

Cdk4, Cdk6, and Cdk2 are clients of HSP90 (see the website http://www.picard.ch for details), and are reported as being reduced by HSP90 inhibitors [17,18]. We thought the reductions of these Cdks in our model might also be related to inhibition of HSP90, a recently identified effect that celastrol has on different cell lines [11,12,21]. To support this line of thought, down-regulation of these Cdks of our model was accompanied by strong expression of HSP70, a telltale sign of HSP90 inhibition. HSP90 inhibition was also confirmed by celastrol's reduction of ATPase activities in the HSP90 immunoprecipitated complex, a finding that agrees with the latest report [28]. Further supporting our hypothesis, co-immunoprecipitation showed reductions in Cdk4 or Cdk6 to HSP90 combinations. These findings are consistent with previous reports describing celastrol's effective disruption of HSP90-client-protein interactions and down-regulation of HSP90 clients such as androgen receptors (AR), Akt, epidermal growth factor receptors (EGFR), etc [11,12,29]. It is worth noting that celastrol's reduction of Cdk4 has also been observed in other cell types [12], so it may be celastrol's general action to affect this cell cycle regulator, a hypothesis that needs further elucidation. Though Cyclin D1 is not a confirmed HSP90 client, its reduction could be explained as a result of HSP90 inhibition. Cyclin D1 expression is controlled by multiple signaling pathways, some key kinases of which are affected by HSP90 [30-32]. Several of these kinases have been proven to be affected by celastrol [24,29,33,34]. Nevertheless, direct interaction of celastrol and HSP90 has not been demonstrated *in vivo*, and due to the reactive nature of celastrol it is possible that it might have additional targets which contribute to a stress response in the cells.

Another of our important findings is that celastrol's actions upon cell cycle and HSP90 clients can be reversed by pre-treatment with thiol-containing agents, such as NAC or GSH, but not by GSSG or non-thiol reducing agents like Vit C. Our findings agree with previous investigations by Trott *et al*, who reported that celastrol induced heat shock response- and antioxidant response-inducible transcripts in RKO human colorectal carcinoma cells, these effects decreasing upon incubation with 250 μM DTT [9]. These results, when viewed together, indicate that thiol can reverse celastrol's actions.

To explore the possible mechanism for thiol-containing agents' reversing effects toward celastrol, we investigated the possibility of direct reaction between these agents and celastrol. Free-thiol containing agents caused celastrol's colour to fade, while non-thiol agents did not give this primary evidence of direct reaction. Absorbance spectrum analysis and MS detection provided further evidence that celastrol was able to bind with thiol-containing agents but not with non-thiol molecules. It has been shown that celastrol can react or bind with some proteins and truncated proteins [12,21,34], this combination achieved either by direct reactions of celastrol with cysteine residues or by inserting celastrol into the 'pockets' related to or affected by cysteine residues. Here we find convincing evidence that celastrol directly reacts with thiol. Since the thiol-containing agents we tested, such as NAC and GSH, are small molecules and thus non-'pocket' forming (as seen in the 3D structure of large molecules), the bonds they form should be a direct chemical reaction between celastrol and the thiol group. Our results agree with the predication that the electrophilic sites within celastrol's A and B rings could react with nucleophilic groups of amino acid residues to form covalent Michael adducts [35]. Sreeramulu *et al *recently showed that H6 on celastrol's ring B might be the reactive site [21], a finding further confirmed by our tests (see additional file 3).

Therefore, the reversing effects of thiol-containing agents might be achieved using either one or both of the following ways. The first way is by bonding the thiol-containing agents (we used) to celastrol to reduce pharmacological effect. Thiol modifications of this type are not rare when reducing a drug's biological actions-- for example, NAC reportedly forms a complex with geldanamycin to reduce the latter's HSP90 inhibiting activities [36]. The second way is that thiol-containing agents added to cells compete with the cellular thiols targeted by celastrol, thus lessening celastrol's overall impact on cellular thiols. Each year in China, there are reports of death caused by the toxicity of celastrol-containing preparations. The reversing effects of thiol-containing agents indicated in these models might be used for relief of such instances of celastrol-caused toxicity.

Direct thiol reaction also provided us with a new understanding of celastrol-caused HSP90 inhibition. The HSP90 chaperone complex consists of proteins that contain the cysteine residues that are celastrol's potential targets. Of several cysteines in HSP90's middle and C-terminals, at least one of them is important to HSP90 activity [37], and it cannot be ruled out that these are attacked by celastrol. In fact, HSP90's C-terminal reaction to celastrol was recently confirmed by Zhang *et al*. [28]. (We also found preliminary evidence that celastrol could directly reduce the number of reactive thiols in recombinant human HSP90α *in vitro*). In addition to HSP90 itself, cysteines in co-chaperones, especially Cdc37, might be attacked by celastrol. Concurrent with the preparation of this manuscript, Sreeramulu *et al*. reported that celastrol could directly combine with Cdc37 in a cysteine-dependent way [21]. Our results, like previous reports [12], showed disruption of the HSP90/Cdc37 complex, likely based on celastrol's effects upon Cdc37. More generally, HSP90's clients and other regulators of HSP90 with cysteines are all possible targets of celastrol. In following with this consideration, the activities of HSP90's clients IKK, proteasome, and ERK were directly inhibited when tested in a cell-free system [15,29,34].

Celastrol's reaction with thiol also suggests that in addition to the HSP90 chaperone complex, other proteins may be affected by celastrol. To support this supposition, topoisomerase II, tubulin, and full-length mutant huntingtin are all reported to directly react with celastrol in a cell-free system [14,38,39]. The more proteins celastrol affects, the greater the chance for side effects. It is reasonable to consider that the spectrum of proteins targeted by celastrol will become broader as dose increases. This is because at low doses celastrol may only affect proteins with rich content, but at high doses low-level proteins may also be affected. Since HSP90 is one of the richest proteins and has elevated expressions in tumor cells, we would recommend low dosage application of this agent to limit side effect risks.

## Conclusions

We found that celastrol could arrest human monocytic leukemia cells U937 at G0/G1 phase, this arrest accompanied by down-regulation of Cyclin D1, Cdk4, Cdk6, and Cdk2. This not only reveals a new action of celastrol, but also suggests its possible application in leukemia and atherosclerosis (a highly prevalent disease related to abnormal monocyte proliferation) treatment. Direct reactions between celastrol and thiol also shed new light on the action of this HSP90 inhibitor providing a useful strategy for relieving celastrol's toxicity. The target spectrum for celastrol, however, might be dose-dependent. The answer to such a question will ultimately need further investigation to provide a more detailed application basis for this anti-proliferation agent.

## Methods

### Reagents and drugs

RPMI 1640 medium, fetal bovine serum (FBS) and streptomycin/penicillin for cell culture were obtained from PAA Laboratories (Linz, Austria). Propidium iodide (PI), dimethyl sulfoxide (DMSO) and protease inhibitor cocktail were purchased from Sigma (St. Louis, MO). 7-Amino-actinomycin D (7-AAD) was purchased from Anaspec (San Jose, CA), carboxyfluorescein diacetate, succinimidyle ester (CFSE) was purchased from Molecular Probe (Eugene, OR). 17-allylamino-17- demethoxygeldanamycin (17-AAG) was obtained from Invivogen (San Diego, CA). N-acetylcysteine (NAC), reduced glutathione (GSH), oxidized glutathione (GSSG) and dithiothreitol (DTT) were products of Amersco (Solon, OH). ATP, NADH and pyruvate kinase (PK) were obtained from BBI (distributed by Guan Yu Co., Shanghai, China). Vitamin C (Vit C), L-lactate dehydrogenase (L-LDH) and phosphoenolpyruvate (PEP) were obtained from Sigma (St. Louis, MO). Protein A/G plus agarose was obtained from Santa Cruz Biotechnology (Santa Cruz, CA). Anti-Cyclin D1 antibody was purchased from Zymed (South San Francisco, CA). Anti-Cdk4 and Cdk2 antibodies were purchased from BD Biosciences (San Jose, CA). Anti-Cdk6 and Cdc37 antibodies were obtained from Santa Cruz Biotechnology (Santa Cruz, CA). Anti-HSP70 was purchased from USBiological (Swampscott, Massachusetts). Anti-HSP90 (H9010) for co-immunoprecipitation was purchased from Alexis Biochemicals (San Diego, CA), and anti-HSP90 (SPA-830) for western blot was obtained from Stressgen Bioreagents (Ann Arbor, MI). Anti-actin antibody, BCA protein assay reagent kit and Beyo ECL Plus for western blot were purchased from Beyotime Biotechnology (Jiangsu, China). All reagents were stored as recommended by the manufactures.

Celastrol was extracted as previously reported by us [33]. Celastrol was dissolved in 50 mM in DMSO and stored at -20°C to be used within 3 months after preparation. The stored solution was further diluted with RPMI 1640 medium to a proper lower concentration immediately before experiments.

### Cell culture and treatment

Human monocytic leukemia cell line U937 was obtained from the Shanghai Cell Bank of the National Science Academy of China (Shanghai). Cells were maintained in RPMI 1640 supplemented with 10% FBS, 100 IU/ml penicillin and 100 μg/ml streptomycin in a humidified 5% CO_2 _incubator at 37°C. Exponentially growing cells were used for experiments. Cells were seeded into 96-well or 24-well culture plates or 100 mm culture dishes at a density of 2 × 10^5^/ml followed by exposure to indicated doses of celastrol for an indicated time. The culture medium with DMSO (vehicle) served as celastrol's control. The final concentration of DMSO never exceeded 0.1%. Each experiment was repeated at least three times.

### Cell counting

At the end of indicated time points, cells were collected and the living and dead cells enumerated. Accurate enumeration of living and dead cells was carried out by FCM based on a single-tube platform with self-made cell-Beads as internal controls, a method originally reported by Harrison *et al *[19] and modified by us [20]. Briefly, after samples were washed with PBS, a known number of green fluorescence-containing Cell-Beads were added. Before analysis by FACScalibur flow cytometer (Becton-Dickinson, CA), PI with a final concentration of 1 μg/ml was added. The FL1 flow cytometric detector was used for discrimination between Cell-Beads and U937 cells, based on the signal of green fluorescence which was positive for Cell-Beads but not for U937. The FL2 detector was used to discriminate the living cells from the dead, which tested negative and positive for PI's signal respectively. The total events detected were 10,000. The number of living (or dead) U937 cells was calculated using the following equation:

The Cell-Beads in our experiments were created by labeling THP-1 cells with CFSE according to the manufacturer's recommended protocol. CFSE-labeled cells were fixed with 1% paraformaldehyde and washed with PBS. CFSE-labeled cells were stored at 4°C.

### Cell cycle analysis

Following cold PBS wash, cells were fixed in 70% ice-cold ethanol for 1 h. The samples were incubated in 50 μg/ml RNase A and 25 μg/ml PI for 30 min at 37°C. The DNA contents of more than 15,000 cells were detected by FCM. Quantitative analysis of cell cycle distribution was performed using ModFit LT Macintosh software (Verity Software House, Inc., ME).

### Apoptosis detection

Apoptotic cells were assessed using Annexin V- fluorescein isothiocynate (FITC) and PI double staining kit (Jingmei biotechnology, China) according to the manufacturer's instructions. Briefly, after being washed twice with cold PBS, Cells were incubated in 100 μl binding buffer containing 5 μl Annexin V-FITC and 10 μl PI (20 μg/ml) for 15 min at room temperature in the dark. Apoptotic cells were analysed by FCM.

### Flow cytometric detection of protein expression

Following being washed with PBS, cells were fixed in 100% methanol for 10 min at 4°C, and then incubated in the indicated primary antibodies for 45 min at 4°C, with appropriate isotypes as control, following by the corresponding secondary antibodies in conjunction with PE or FITC for 30 min at 4°C. The samples were analyzed by FCM. The analyses were performed with CellQuest software (BD Biosciences). For samples that required for simultaneous detection of proteins and cell cycle, cells were subjected to RNA degradation (as mentioned above) and DNA staining with 7-AAD after the secondary antibody labeling.

### Co-immunoprecipitation and western blot

Cells were incubated in lysis buffer (20 mM Tris·HCl, 25 mM NaCl, 0.1% NP40, 2 mM DTT, 20 mM Na_2_MoO_4_, and protease inhibitor cocktail, pH 7.4) for 2 h at 4°C, and lysates were cleared by centrifugation at 13,000 × g for 10 min. Protein concentrations were determined by BCA protein assay reagent kit. 1 mg of proteins were incubated with 2 μg of anti-HSP90 (H9010) antibody overnight at 4°C, and then 30 μl Protein A/G plus agarose was added for additional 3 h at 4°C. Beads were washed three times with PBS and diluted in 5 × SDS-sample buffer and heated to 95°C for 5 min.

Aliquots of samples were loaded onto 10% SDS-polyacrylamide gels and then transferred to polyvinylidene difluoride (PVDF) membranes. Membranes were probed with indicated antibodies. Detection was accomplished using corresponding horseradish peroxidase (HRP)-conjugated secondary antibodies followed by development with Beyo ECL Plus and autoradiography with film.

### ATPase activity assay

Untreated cells were co-immunoprecipitated using anti-HSP90 (H9010) antibody as described above. Beads bound to the immunoprecipites were washed and separated into three equals portions. Each portion of beads was then combined with either 0.06 mM of celastrol, 0.6 mM of 17-AAG, or 0.6 mM of DMSO at 37°C for 10 min. The ATPase activity assay is based on a regenerating coupled enzyme assay [22], in which the phosphorylation of ADP during the catalyzation of phosphoenolpyruvate (PEP) by pyruvate kinase (PK) is coupled to the reduction of the resulting pyruvate by lactate dehydrogenase (LDH) at the expense of NADH. Oxidation of NADH to NAD^+ ^produced an absorbance decrease at 340 nm. Each 250 μl assay contained 100 mM Tris-HCl (pH 7.4), 20 mM KCl, 6 mM MgCl_2_, 0.8 mM ATP, 0.1 mM NADH, 2 mM PEP, 0.2 mg PK, and 0.05 mg L-LDH. Following incubation, drug-treated beads were added into the reaction buffer. ATPase activity was detected as decreasing in absorbance at 340 nm.

### Reaction between celastrol and free-thiol containing agents *in vitro*

NAC, GSH, GSSG, DTT, or Vit C was added into 1 ml of celastrol at 280 mM with a molecular ratio of 2:1, respectively. The mixtures were then left at room temperature for 30 min. The absorption spectra of the mixtures were measured with an ultraviolet visible spectrophotometer (UV757CRT, Shanghai Precision and Scientific Instrument Co., Shanghai). The spectrums of celastrol alone and of each reactant added alone were measured as control. To further study the reaction between celastrol and thiol (non-thiol)-containing agents, celastrol was mixed with these agents in a molar ratio of 1:2, respectively, in DMSO for at least 20 min. The samples were analyzed by Q-Tof micro YA019 mass spectrometer (Waters Corp. USA).

### Statistical analysis

Data are presented as mean ± SD. One-way analysis of variance (ANOVA) was used for statistical evaluation of significant differences among the groups using SPSS 11.5 for Windows software. A value of *P *< 0.05 was considered to be statistical significance. Experiments were repeated at least three times.

## Competing interests

The authors declare that they have no competing interests.

## Authors' contributions

BP carried out the immunoassays, participated in the design of the study, performed the statistical analysis and drafted the manuscript. LX participated in cell culture. FC carried out cell counts. TW participated in design of the study. CY carried out celastrol extraction. GU participated in study design and helped to draft the manuscript. DZ conceived of the study, participated in its design and coordination, and helped to draft the manuscript. All authors have read and approved the final manuscript.

## Supplementary Material

Additional file 1 **HSP90 ATPase assay based on inorganic phosphate released by ATP hydrolysis**. In addition to using a regenerating coupled enzyme assay to determine the effects of celastrol on ATPase in HSP90, we also tested this enzyme's activity by another type of assay, based on the inorganic phosphate released by ATP hydrolysis (Song *et al*, J Biol Chem, 2003, 278: 3648-3655). The results showed that celastrol and 17-AAG could inhibit ATPase activity in the HSP90 complex. Click here for file

Additional file 2 **MS analysis of the reactive products between the free thiol-containing agents and celastrol**. MS analysis showed that all thiol-containing agents ((NAC, GSH or DTT) could react with celastrol and form an addition product, while GSSG and reducing agent Vit C did not accomplish this. These reactions between free thiol-containing agents and celastrol were reversible, as confirmed by the disappearance of the addition product peak and reappearance of the celastrol peak. Click here for file

Additional file 3 ^**1**^**H NMR spectrum analysis the reactive site on celastrol**. Celastrol was mixued with NAC at a ratio of 1:2 in DMSO-d_6 _and 1D ^1^H NMR spectrum were recorded, the results showed the Michael adduct at C6 in ring B of celastrol, as previously reported by Sreeramulu S [21]. Click here for file
