# Supplementary material for: HSP90 inhibitor, celastrol, arrests human monocytic leukemia cell U937 at G0/G1 in thiol-containing agents reversible way
Source: Mol Cancer. 2010 Apr 16;9:79. doi: 10.1186/1476-4598-9-79 (PMC2873437; doi:10.1186/1476-4598-9-79)
Supplement: Additional file 1 — HSP90 ATPase assay based on inorganic phosphate released by ATP hydrolysis. In addition to using a regenerating coupled enzyme assay to determine the effects of celastrol on ATPase in HSP90, we also tested this enzyme's activity by another type of assay, based on the inorganic phosphate released by ATP hydrolysis (Song et al, J Biol Chem, 2003, 278: 3648-3655). The results showed that celastrol and 17-AAG could inhibit ATPase activity in the HSP90 complex. [file 1476-4598-9-79-S1.PDF]

Additional file 1

HSP90 ATPase assay based on inorganic phosphate released by ATP hydrolysis

In addition to using a regenerating coupled enzyme assay to determine the effects of celastrol on ATPase in HSP90, we also tested this enzyme's activity by another type of assay, based on the inorganic phosphate released by ATP hydrolysis (Song *et al*, J Biol Chem, 2003, 278: 3648-3655).

Intact U937 cells were co-immunoprecipitated by the H9010 antibody as detailed in the Methods section. Beads bound to the immunoprecipitates were washed in IP lysis buffer and separated into three equal portions. Each 10 μl of beads were then incubated with 5 μl of 0.125 mg/ml of celastrol, 1 mg/ml of 17-AAG, or DMSO at room temperature for 10 min. ATPase activity assay was performed according to the instructions of ATPase activity assay kit (Jiancheng Bio Co., Nanjing, China), which is based on the inorganic phosphate released by ATP hydrolysis. The absorbance at 630 nm was measured using Multiskan MK3 Microplate Reader (Thermo Electron Instrument). The results showed that the celastrol and 17-AAG could inhibit ATPase activity in the HSP90 complex (Figure S1)

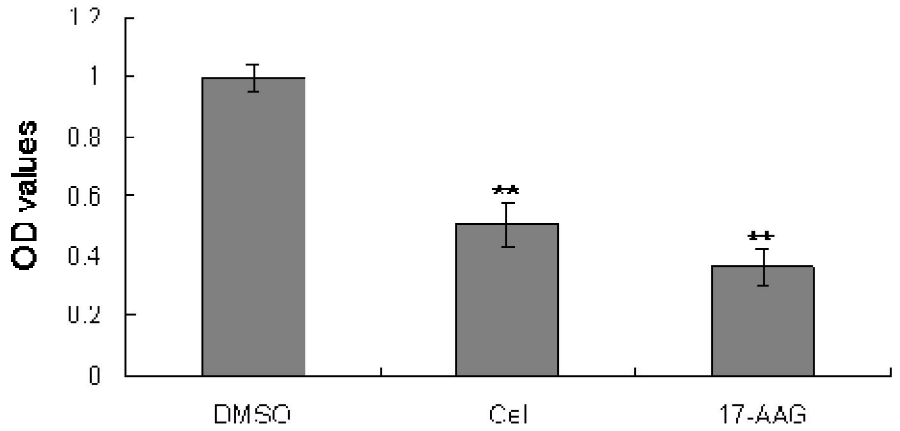

Figure S1. Inhibition of ATPase activity in HSP90 complex pulled down by IP. OD value on Y-axis means OD value relative to the DMSO control; ** represents $P < 0.01$ compared with the DMSO control. n =3.
