# Supplementary material for: HSP90 inhibitor, celastrol, arrests human monocytic leukemia cell U937 at G0/G1 in thiol-containing agents reversible way
Source: Mol Cancer. 2010 Apr 16;9:79. doi: 10.1186/1476-4598-9-79 (PMC2873437; doi:10.1186/1476-4598-9-79)
Supplement: Additional file 2 — MS analysis of the reactive products between the free thiol-containing agents and celastrol. MS analysis showed that all thiol-containing agents ((NAC, GSH or DTT) could react with celastrol and form an addition product, while GSSG and reducing agent Vit C did not accomplish this. These reactions between free thiol-containing agents and celastrol were reversible, as confirmed by the disappearance of the addition product peak and reappearance of the celastrol peak. [file 1476-4598-9-79-S2.PDF]

Additional file 2

MS analysis of the reactive products between the free thiol-containing agents and celastrol

The reactive product between thiol-containing agents and celastrol was tested by MS analysis. The results showed that all thiol-containing agents ((NAC, GSH or DTT) could react with celastrol and form an addition product, while GSSG and reducing agent Vit C did not accomplish this. These reactions between free thiol-containing agents and celastrol were reversible, as confirmed by the disappearance of the addition product peak and reappearance of the celastrol peak when detected by MS. The following (Figure S2) is the detection pattern of the reaction between DTT and celastrol. A truncated version of this figure is included in the manuscript.

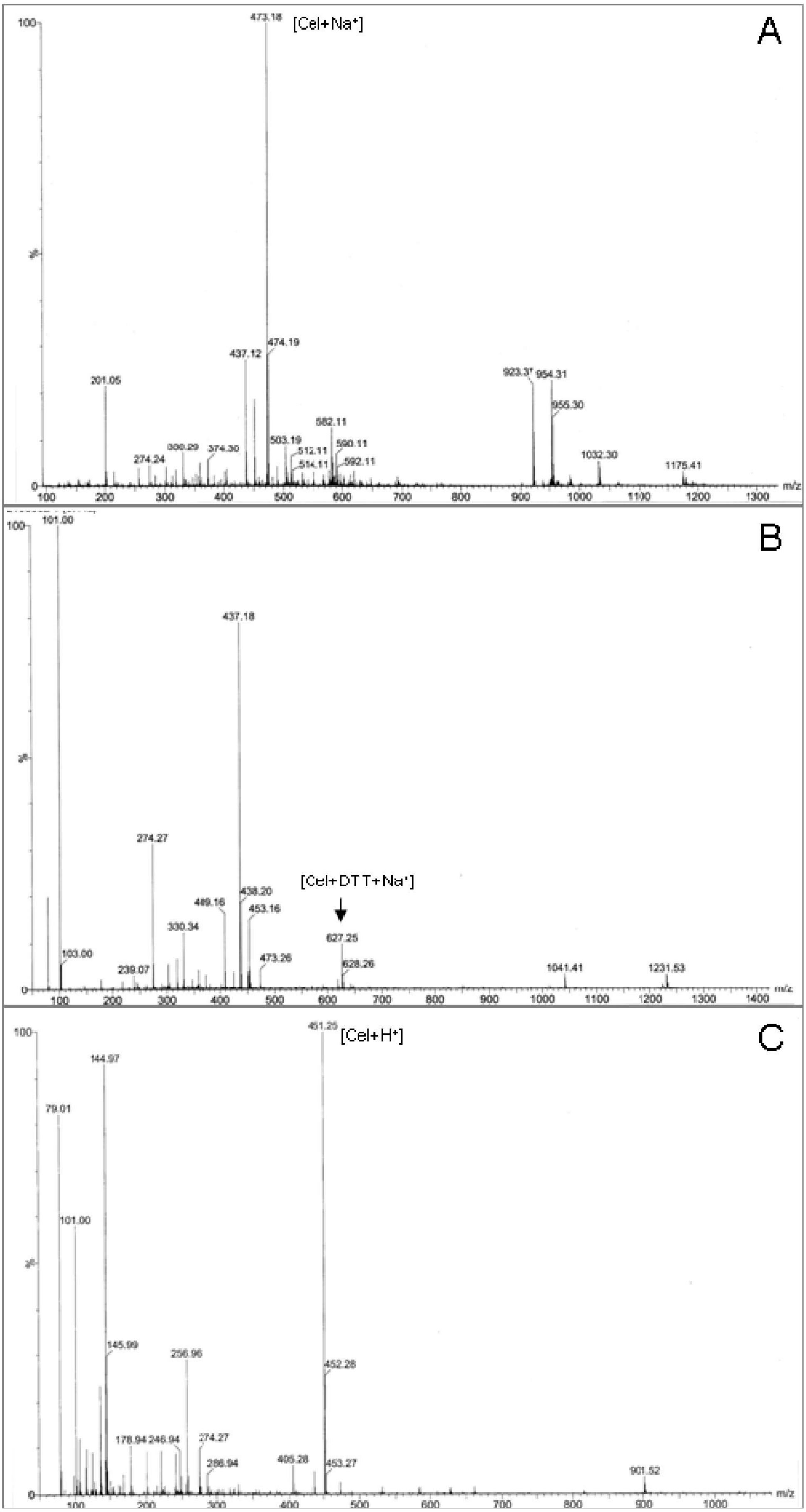

[Cel+Na⁺]
A
473.18
474.19
437.12
201.05
923.31
954.31
955.30
582.11
503.19
590.11
592.11
374.30
330.29
274.24
1032.30
1175.41
B
101.00
437.18
274.27
[Cel+DTT+Na⁺]
409.16
438.20
453.16
330.34
627.25
628.26
103.00
473.26
239.07
1041.41
1231.53
C
451.25
[Cel+H⁺]
144.97
79.01
101.00
145.99
256.96
452.28
178.94
246.94
274.27
286.94
405.28
453.27
901.52
m/z
%

Figure S2. MS detection of the addition product of DTT with celastrol. **A**: celastrol in DMSO, m/z 473.18 represents celastrol (binding to one natrium). **B**: celastrol mixed with DTT, m/z 627.25 represents addition product of celastrol and DTT (binding to one natrium). **C**: addition of formic acid into the reaction system of DTT and celastrol. m/z 451.25 represents celastrol's reappearance (binding to one hydrogen).
