# Supplementary material for: HSP90 inhibitor, celastrol, arrests human monocytic leukemia cell U937 at G0/G1 in thiol-containing agents reversible way
Source: Mol Cancer. 2010 Apr 16;9:79. doi: 10.1186/1476-4598-9-79 (PMC2873437; doi:10.1186/1476-4598-9-79)
Supplement: Additional file 3 — 1H NMR spectrum analysis the reactive site on celastrol. Celastrol was mixued with NAC at a ratio of 1:2 in DMSO-d6 and 1D 1H NMR spectrum were recorded, the results showed the Michael adduct at C6 in ring B of celastrol, as previously reported by Sreeramulu S [21]. [file 1476-4598-9-79-S3.PDF]

Additional file 3

^{1}H NMR spectrum analysis the reactive site on celastrol

To find out the location of celastrol's addition reaction, we mixed celastrol with NAC at a ratio of 1:2 in DMSO-d_6 and then recorded 1D ^{1}H NMR spectrum. The spectrum shows the Michael adduct at C6 in ring B of celastrol (Figure S3) as previously reported by Sreeramulu S [21].

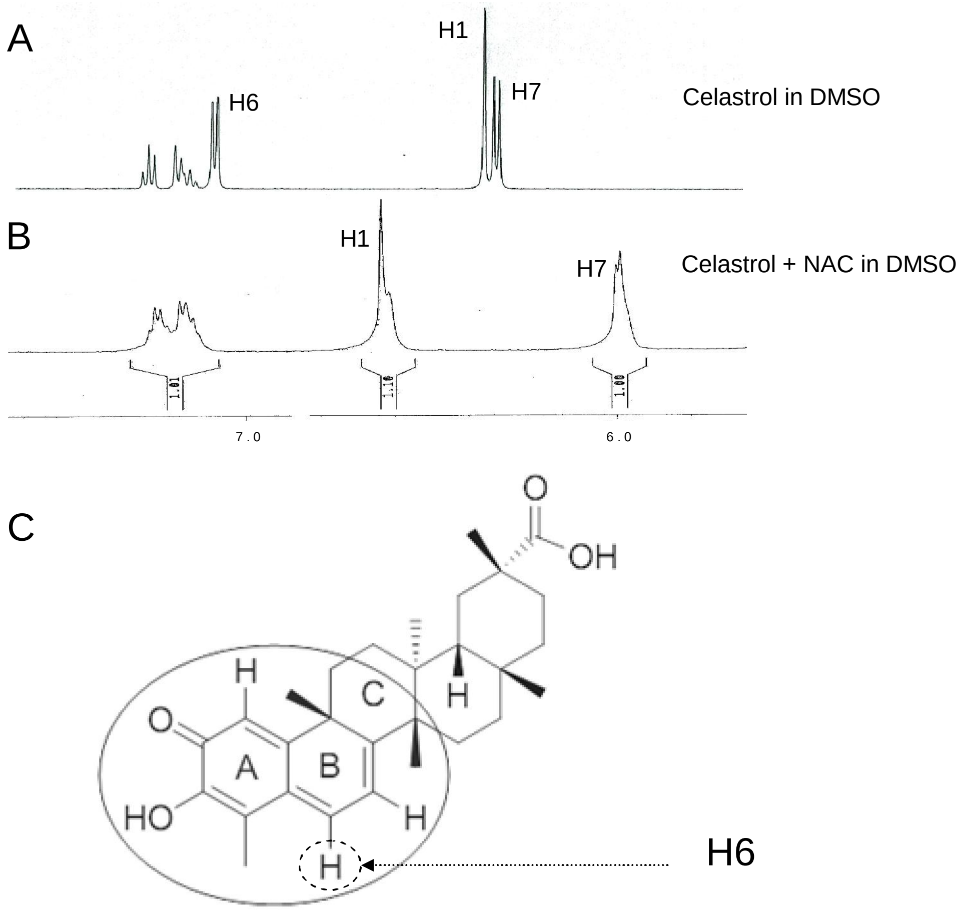

Figure S3. Detection of celastrol's addition reaction site. **A**: the signal detected H1, H6 and H7 on ring A and B in celastrol. **B**: disappearance of H6 on ring B of celastrol when reacted with NAC. **C**: structure of celastrol and H6 in ring B.
